# Preparation of NiS/ZnIn_2_S_4_ as a superior photocatalyst for hydrogen evolution under visible light irradiation

**DOI:** 10.3762/bjnano.4.107

**Published:** 2013-12-23

**Authors:** Liang Wei, Yongjuan Chen, Jialin Zhao, Zhaohui Li

**Affiliations:** 1 Research Institute of Photocatalysis, Fujian Provincial Key Laboratory of Photocatalysis–State Key Laboratory Breeding Base, Fuzhou University, Fuzhou 350002, P. R. China

**Keywords:** co-catalyst, hydrogen evolution, NiS, photocatalytic, photocatalysis, visible light

## Abstract

In this study, NiS/ZnIn_2_S_4_ nanocomposites were successfully prepared via a facile two-step hydrothermal process. The as-prepared samples were characterized by X-ray diffraction (XRD), X-ray photoelectron spectroscopy (XPS), transmission electron microscopy (TEM) and high-resolution transmission electron microscopy (HRTEM). Their photocatalytic performance for hydrogen evolution under visible light irradiation was also investigated. It was found that the photocatalytic hydrogen evolution activity over hexagonal ZnIn_2_S_4_ can be significantly increased by loading NiS as a co-catalyst. The formation of a good junction between ZnIn_2_S_4_ and NiS via the two step hydrothermal processes is beneficial for the directional migration of the photo-excited electrons from ZnIn_2_S_4_ to NiS. The highest photocatalytic hydrogen evolution rate (104.7 μmol/h), which is even higher than that over Pt/ZnIn_2_S_4_ nanocomposite (77.8 μmol/h), was observed over an optimum NiS loading amount of 0.5 wt %. This work demonstrates a high potential of the developing of environmental friendly, cheap noble-metal-free co-catalyst for semiconductor-based photocatalytic hydrogen evolution.

## Introduction

Hydrogen is a clean and green fuel. The conversion and store of solar energy in the form of hydrogen by photocatalytic water splitting holds great promise to meet the future energy and environment requirements [1–3]. Ever since the pioneering work of a photo-electrochemical cell using Pt-TiO_2_ electrodes for water splitting by Fujishima and Honda in 1972, great efforts have been devoted to the development of highly efficient semiconductor photocatalysts for hydrogen production [4]. So far, a variety of active photocatalysts for hydrogen production, including metal oxides [5–8], sulfides [9–11], oxynitrides [12–14], as well as the metal-free semiconductors [15] have already been developed.

Among the numerous types of semiconductor systems studied, metal sulfides have demonstrated promising activities towards hydrogen evolution from water containing sacrificial reagents under visible light. ZnIn_2_S_4_ is a ternary chalcogenide with a suitable band gap (2.34–2.48 eV) well corresponding to the visible light absorption. ZnIn_2_S_4_ exhibits two distinct polymorphs based on cubic and hexagonal lattices [16], which can be controlled synthesized via a facile hydrothermal method using different precursors. Previous studies revealed that both polymorphs of ZnIn_2_S_4_ are active for photocatalytic hydrogen generation under visible light irradiations and show considerable chemical stability [17–19]. However, the photocatalytic hydrogen evolution activity over pure ZnIn_2_S_4_ is low, due to the poor separation efficiency and migration ability of the photo-excited charge carriers. A variety of effects have been made to enhance the photocatalytic performance of ZnIn_2_S_4_. For example, by size control on ZnIn_2_S_4_ [20], doping with transition metals [21] and incorporation of metal sulfides [22] or RGO [23] into ZnIn_2_S_4_ nanostructures, the photocatalytic performance for hydrogen evolution over ZnIn_2_S_4_ have been enhanced to a certain degree.

Studies on semiconductor-based photocatalysts revealed that the deposition of a suitable co-catalyst on the semiconductor photocatalysts can play important roles in promoting their photocatalytic performance for hydrogen evolution. An appropriate co-catalyst can suppress the recombination of the photo-generated charge carriers, lower the overpotential for hydrogen evolution and also provide redox reaction sites for hydrogen evolution to avoid back reactions. Due to their negligible overpotential for hydrogen evolution and excellent kenetics for driving the hydrogen evolution reaction, noble metals like Pt [24–26], Rh [27], Au [28–29] and their oxides like RuO_2_ [30], Rh_x_Cr_2−x_O_3_ [31–33] are generally used as the co-catalysts for photocatalytic hydrogen evolution. However, the precious metals are expensive and to reduce the cost of renewable hydrogen evolution, it is necessary to explore alternative co-catalysts based on inexpensive transition metals.

Our recent studies revealed that MoS_2_, a good electrocatalyst for hydrogen evolution [34], can be an effective co-catalyst in promoting photocatalytic hydrogen evolution over ZnIn_2_S_4_ and MoS_2_/ZnIn_2_S_4_ show even superior performance for hydrogen evolution than Pt/ZnIn_2_S_4_ [35]. NiS, a p-type semiconductor, is also reported to be a good electrocatalyst for cathodic hydrogen evolution in water electrolysis [36]. Although Ni and NiO have already been used as co-catalysts for hydrogen evolution over oxide semiconductor photocatalysts, the application of NiS as co-catalyst for photocatalytic hydrogen evolution is less studied [37–38]. Only until recently, Xu et al. reported that NiS can be used as co-catalyst to enhance the photocatalytic hydrogen evolution over CdS [39]. It was found that NiS/CdS photocatalysts prepared via a simple hydrothermal loading method showed high photocatalytic activity for hydrogen evolution in the presence of lactic acid as sacrificial agent and a high quantum efficiency of 51.3% at 420 nm was obtained in this system. Later on, several other reports have also reported the using of NiS as a co-catalyst for photocatalytic hydrogen evolution [40–42].

Herein, we reported the preparation of NiS/ZnIn_2_S_4_ nanocomposites via a two-step hydrothermal method and its application for photocatalytic hydrogen evolution under visible light irradiation. It was found that NiS can be an effective co-catalyst for photocatalytic hydrogen evolution over ZnIn_2_S_4_. The as-prepared NiS/ZnIn_2_S_4_ nanocomposites showed superior photocatalytic performance for hydrogen evolution under visible light irradiation and the activity of NiS/ZnIn_2_S_4_ with optimized amount of NiS is even higher than that of Pt/ZnIn_2_S_4_. A possible enhancement mechanism based on the co-catalyst and the formed junction for the improved photocatalytic activity in the NiS/ZnIn_2_S_4_ system was also proposed.

## Results and Discussion

**Figure 1:**
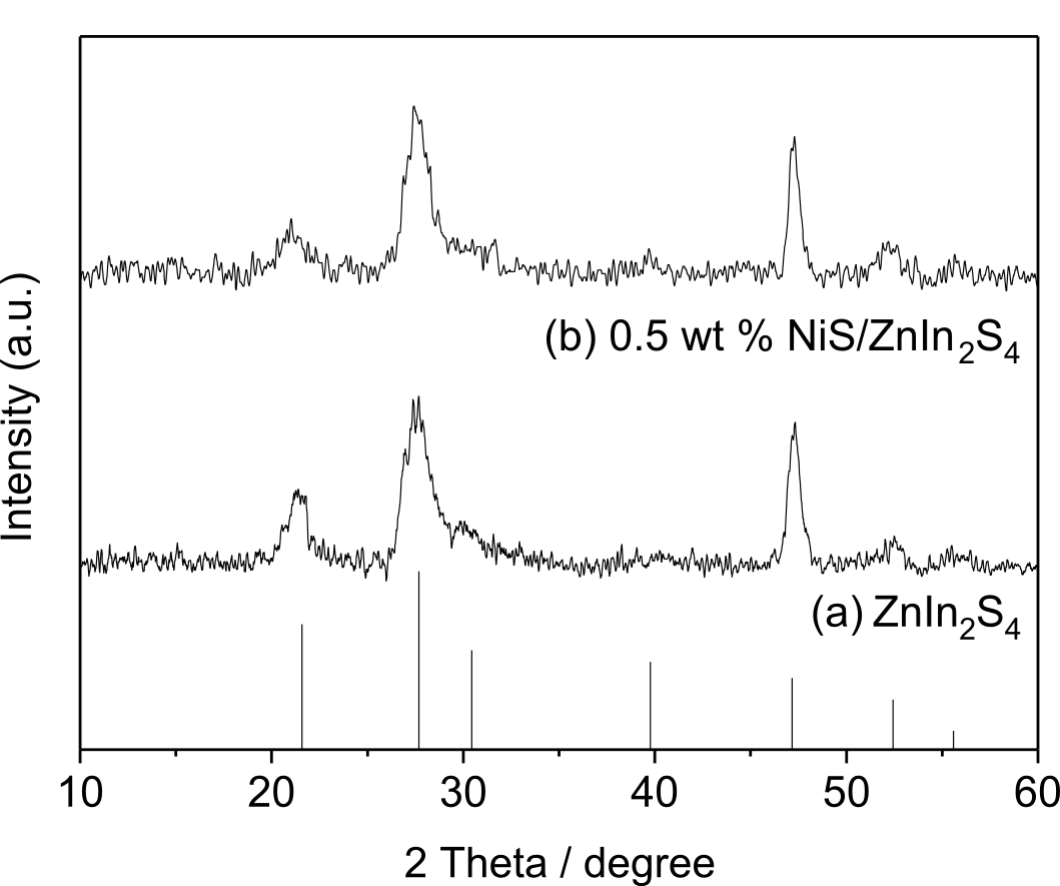
XRD patterns of (a) pure ZnIn_2_S_4_; (b) 0.5 wt % NiS/ZnIn_2_S_4_.

Figure 1 shows the XRD patterns of the as-prepared pure ZnIn_2_S_4_ and sample with loading amount of 0.5 wt % NiS. As shown in Figure 1, all the samples showed diffraction peaks of 2 Theta values at 21.5°, 27.6°, 30.4°, 39.8°, 47.2°, 52.4° and 55.6°, which are corresponding to the (006), (102), (104), (108), (110), (116) and (022) crystallographic planes of hexagonal ZnIn_2_S_4_ (JCPDS, No. 03-065-2023). This suggests that there is no obvious phase change in ZnIn_2_S_4_ during the hydrothermal process in the preparation of the NiS/ZnIn_2_S_4_ nanocomposites. No characteristic diffraction peaks associated with NiS are observed in these samples, probably due to the low amount of NiS loaded and its high dispersion on ZnIn_2_S_4_.

**Figure 2:**
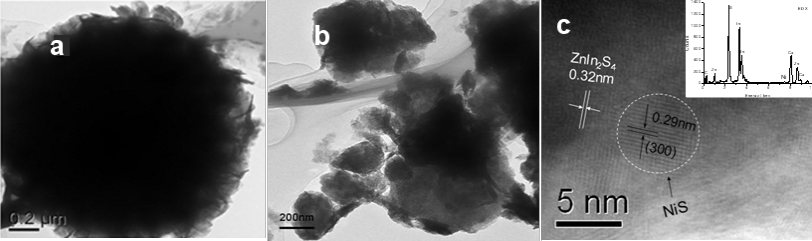
TEM images of (a) ZnIn_2_S_4_; (b) NiS/ZnIn_2_S_4_ and (c) HRTEM image of NiS/ZnIn_2_S_4_ (inset: EDS).

Figure 2a shows the TEM image of our previous studies that the hydrothermally prepared ZnIn_2_S_4_ sample was composed of microspheres with dimension in the range of 2–6 μm assembled by densely packed petals [16]. However, the TEM image of the current NiS/ZnIn_2_S_4_ sample shows that the ZnIn_2_S_4_ microspheres were partially decomposed after the second hydrothermal process (Figure 2b). Although no characteristic diffraction peaks corresponding to NiS nanoparticles were observed in the XRD patterns, the existence of NiS in the nanocomposite is confirmed by the HRTEM image (Figure 2c). Clear lattice fringes of 0.32 nm and 0.29 nm, which can be ascribed to the (102) plane of hexagonal ZnIn_2_S_4_ and the (300) plane of rhombohedral NiS respectively, can be observed. As shown in Figure 2b, the NiS nanoparticle has a diameter of about 5 nm and directly contact with ZnIn_2_S_4_. It is believed that the second hydrothermal treatment during the deposition of NiS on ZnIn_2_S_4_ is important for the formation of a junction between these two components. The energy-dispersive X-ray spectrometry (EDS) analysis as shown in the inset of Figure 2c also confirms the existence of Ni.

**Figure 3:**
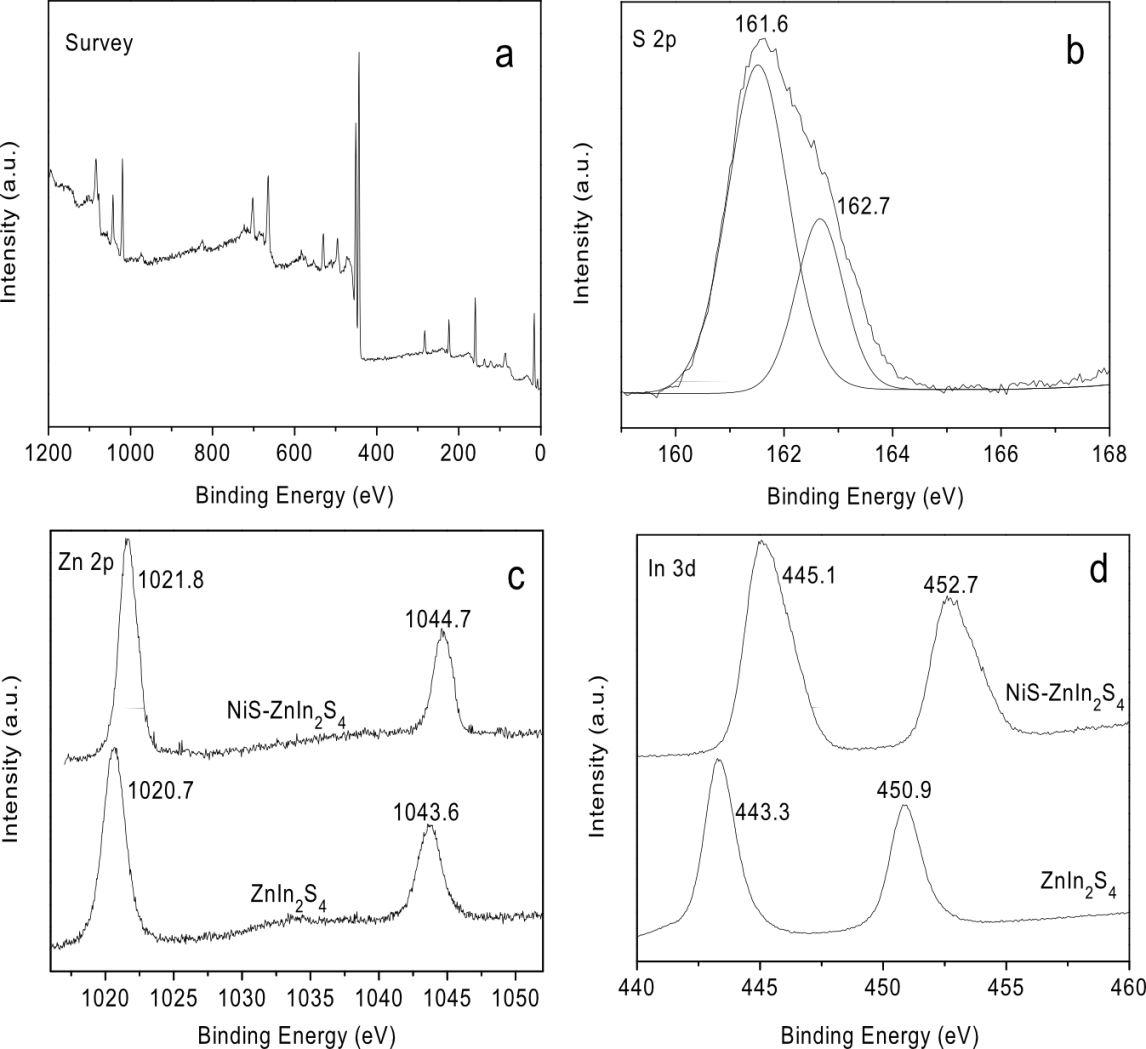
XPS spectra of NiS/ZnIn_2_S_4_ and ZnIn_2_S_4_ (a) survey spectrum and high-resolution spectra for (b) S 2p; (c) Zn 2p and (d) In 3d.

XPS analyses were carried out to evaluate the surface chemical composition and electronic state on 0.5 wt % NiS/ZnIn_2_S_4_ sample, and the results are shown in Figure 3. The survey XPS spectrum, as shown in Figure 3a, confirms the existence of Zn, In, and S. The high resolution XPS spectra of S 2p region can be deconvoluted into two peaks at around 161.7 and 162.8 eV, which can be assigned to S 2p_3/2_ and S 2p_1/2_ respectively in S^2−^ (Figure 3b). As compared to the binding energy of Zn 2p observed over pure ZnIn_2_S_4_ (1020.7 and 1043.6 eV), a higher binding energy shift is observed over NiS/ZnIn_2_S_4_ nanocoposite (1021.8 and 1044.7 eV) (Figure 3c). Similar high binding energy shift is also observed over the high resolution XPS spectra of In 3d (Figure 3d). Such a shift to high binding energy suggests a strong interaction between ZnIn_2_S_4_ and NiS. It is believed that when ZnIn_2_S_4_ are connected to NiS, the electron transfer from ZnIn_2_S_4_ to the more electronegative NiS may result in a decrease of the electron density of Zn^2+^ and In^3+^. Therefore the binding energy of Zn 2p and In 3d shift to a high binding energy in NiS/ZnIn_2_S_4_ nanocoposite. No peaks in the Ni 2p region were observed in the XPS spectra of 0.5% NiS/ZnIn_2_S_4_, probably due to the low amount of NiS loaded and its embed within the ZnIn_2_S_4_ nanostructures.

**Figure 4:**
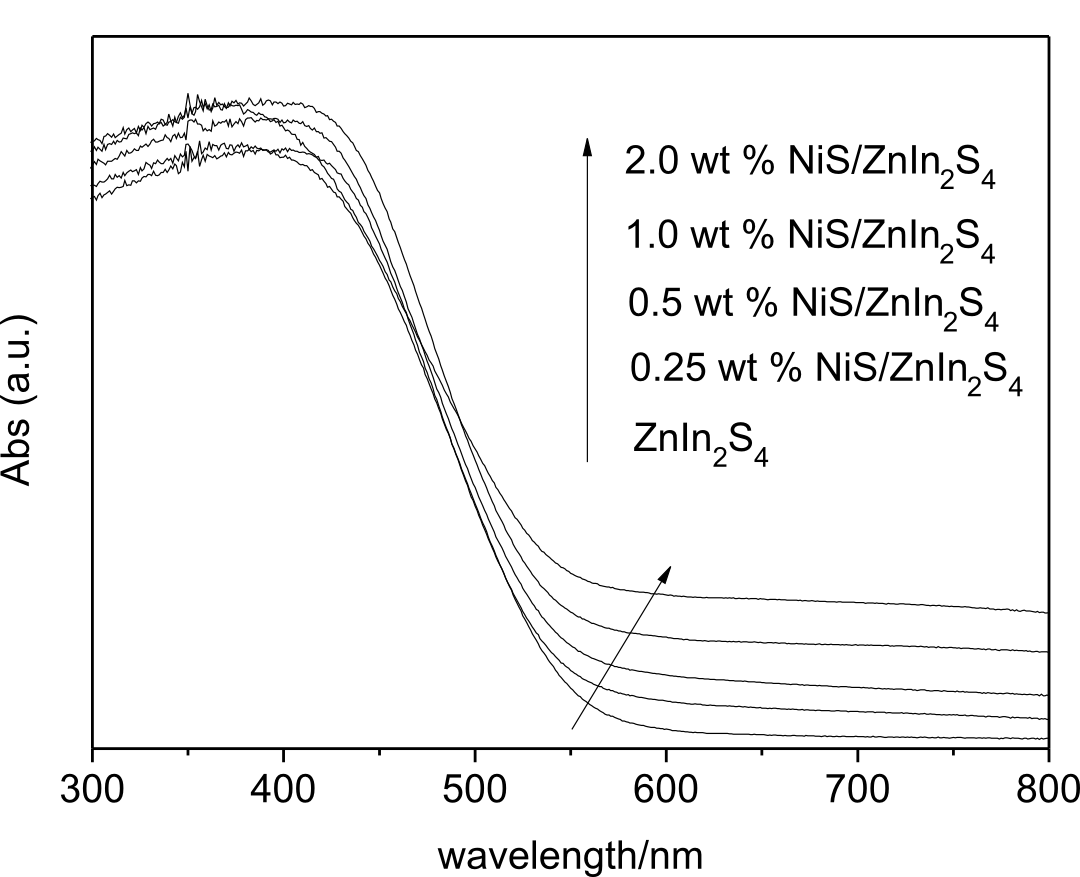
UV–vis diffraction spectra of the pure ZnIn_2_S_4_ and 0.25 wt %, 0.5 wt %, 1.0 wt %, 2.0 wt % NiS/ZnIn_2_S_4_.

The UV–visible diffuse reflectance spectra of NiS/ZnIn_2_S_4_ nanocomposites are displayed in Figure 4. Pure ZnIn_2_S_4_ has an absorption edge at about 540 nm with an energy gap estimated to be 2.4 eV. Although the loading of NiS onto ZnIn_2_S_4_ does not obviously change the band gap of ZnIn_2_S_4_, the resultant NiS/ZnIn_2_S_4_ nanocomposites show an enhanced absorption in the visible light region from 550 to 800 nm, attributable to the absorption of NiS.

**Figure 5:**
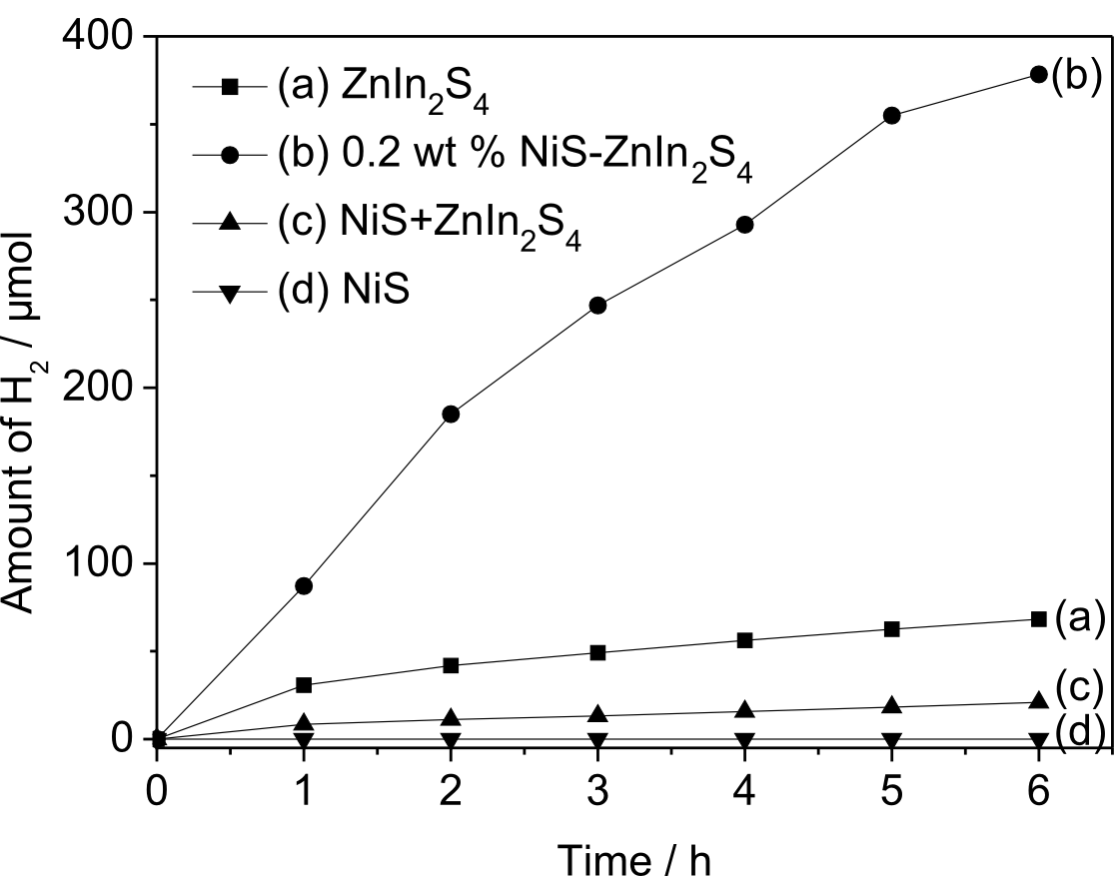
Amount of hydrogen evolution over (a) pure ZnIn_2_S_4_; (b) 0.2 wt % MoS_2_/ZnIn_2_S_4_; (c) mechanical mixture of NiS + ZnIn_2_S_4_ and (d) NiS (Reaction conditions: catalyst, 0.05 g; 100 mL H_2_O containing 0.43 M Na_2_S and 0.5 M Na_2_SO_3_).

Photocatalytic hydrogen evolution experiments were carried out over the as-prepared NiS/ZnIn_2_S_4_ nanocomposites in the presence of Na_2_S/Na_2_SO_3_ as sacrificial agent under visible light irradiation. Figure 5 shows the amount of hydrogen evolved over 0.2 wt % NiS/ZnIn_2_S_4_ nanocomposite and is compared with that of pure ZnIn_2_S_4_ and NiS. Although NiS is a good electrocatalyst for hydrogen evolution [36], no hydrogen was evolved when NiS alone was used as the photocatalyst. Pure ZnIn_2_S_4_ only had a very low activity with the hydrogen evolution at a rate of 14.1 μmol/h. However, the doping of only 0.2 wt % NiS onto ZnIn_2_S_4_ led to its highly enhanced photocatalytic activity for hydrogen evolution. The hydrogen evolution rate over 0.2 wt % NiS/ZnIn_2_S_4_ was enhanced to 70.5 μmol/h, about 5 times of that over pure ZnIn_2_S_4_ under similar condition. This indicates that NiS deposited on the surface on ZnIn_2_S_4_ can significantly promote the photocatalytic hydrogen evolution over ZnIn_2_S_4_.

**Figure 6:**
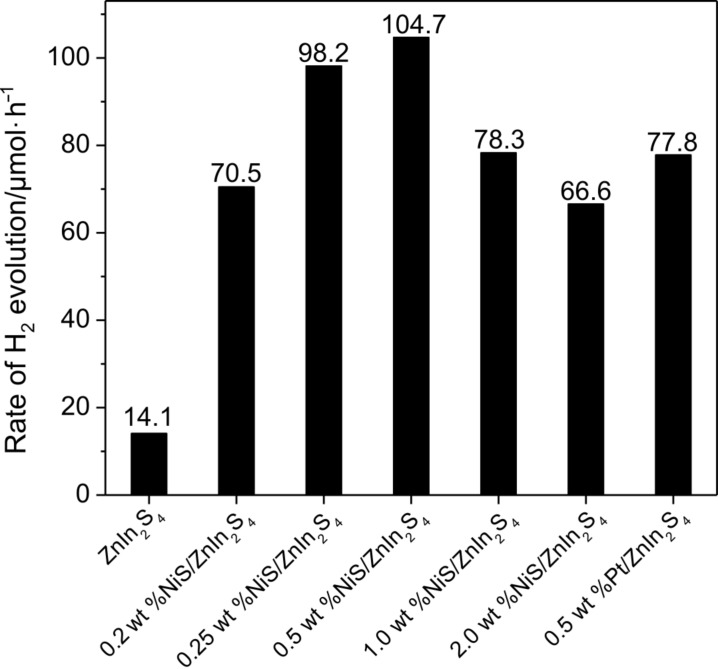
Photocatalytic hydrogen evolution rate over pure ZnIn_2_S_4_; ZnIn_2_S_4_ with different amounts of NiS: 0.2 wt %; 0.25 wt %; 0.5 wt %; 1.0 wt %; 2.0 wt % and 0.5 wt % Pt/ZnIn_2_S_4_. (Reaction conditions: catalyst, 0.05 g; 100 mL H_2_O containing 0.43 M Na_2_S and 0.5 M Na_2_SO_3_).

The effect of the amount of NiS loaded on the photocatalytic performance for hydrogen evolution over ZnIn_2_S_4_ has also been investigated and the results are shown in Figure 6. With increasing the amount of loaded NiS on ZnIn_2_S_4_, the rate of hydrogen evolution on 0.25 wt % NiS/ZnIn_2_S_4_ was enhanced to 98.2 μmol/h, about 7 times as that over pure ZnIn_2_S_4_. An optimum NiS loading amount is found at 0.5 wt %, which exhibits the highest photocatalytic hydrogen evolution rate of 104.7 μmol/h, almost 7.4 times as high as that obtained over the NiS-free ZnIn_2_S_4_. This value is much higher than that observed over 0.5 wt % Pt/ZnIn_2_S_4_ nanocomposite (77.8 μmol/h). A further increase in the amount of NiS resulted in a decrease in the photocatalytic hydrogen evolution rate. Such a decrease in the activity of samples with a heavy loading of NiS is likely due to the shading effect of NiS, which can block the absorption of the incident light by ZnIn_2_S_4_. Therefore, an appropriate loading amount of NiS is crucial to achieve the optimized photocatalytic activity of the ZnIn_2_S_4_ photocatalyst.

**Figure 7:**
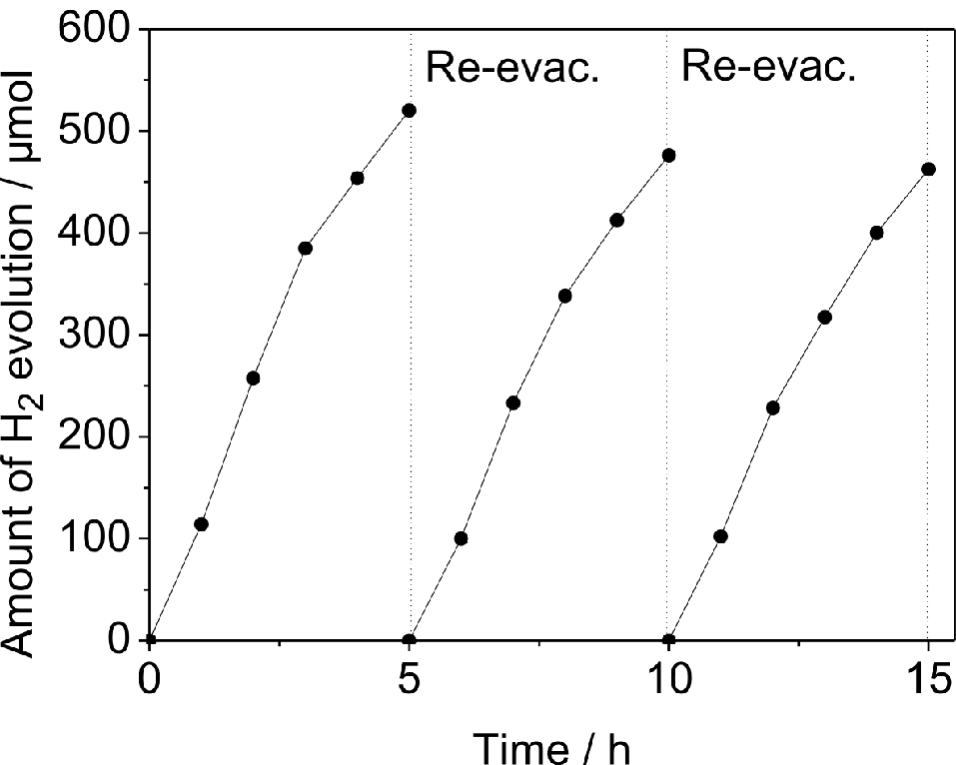
Amount of hydrogen evolved over 0.5 wt % NiS/ZnIn_2_S_4_ system in a 15 h photocatalytic reaction. (Reaction conditions: catalyst, 0.05 g; 100 mL H_2_O containing 0.43 M Na_2_S and 0.5 M Na_2_SO_3_).

**Figure 8:**
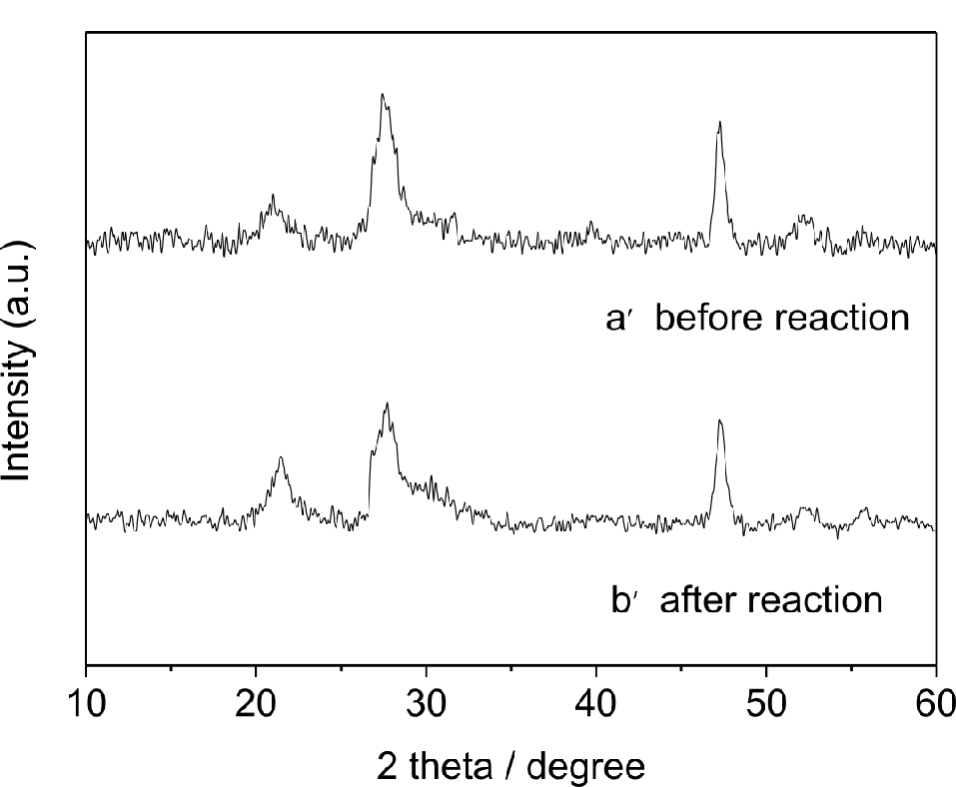
XRD patterns of 0.5 wt % NiS/ZnIn_2_S_4_ (a) before and (b) after photocatalytic hydrogen evolution reaction.

NiS/ZnIn_2_S_4_ nanocomposites show high stability during the photocatalytic hydrogen evolution reaction. A prolonged photocatalytic reaction for 15 h over 0.5 wt % NiS/ZnIn_2_S_4_ revealed that no obvious loss of the activity during the whole reaction period (Figure 7). Besides this, the unchanged XRD pattern of the photocatalyst after the long time reaction also confirms its high stability (Figure 8).

**Scheme 1:**
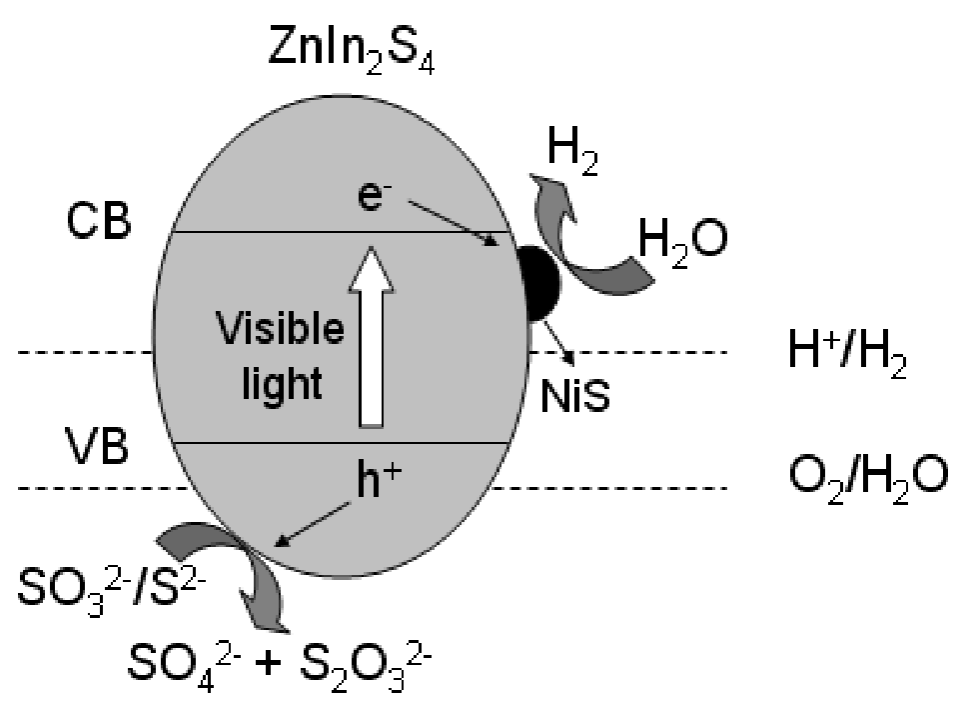
Schematic illustration of proposed mechanism for photocatalytic hydrogen evolution over NiS/ZnIn_2_S_4_ nanocomposite under visible light irradiations.

Scheme 1 shows the mechanism proposed for the enhanced photocatalytic hydrogen evolution over NiS/ZnIn_2_S_4_ nanocomposite. Although the conduction band edge of ZnIn_2_S_4_ (−1.1 eV) is higher than the reduction potential of H^+^/H_2_, the rate of hydrogen evolution is low over bare ZnIn_2_S_4_ due to the rapid recombination rate of photogenerated charge carriers as well as the presence of a large hydrogen evolution overpotential. When NiS is deposited on the surface of ZnIn_2_S_4_, due to the less negative conduction band of NiS as compared to that of hexagonal ZnIn_2_S_4_ [43], a directional transfer of the photogenerated electrons from the conduction band of ZnIn_2_S_4_ to NiS is feasible. Since NiS is a good electrocatalyst for hydrogen evolution [36], NiS can adsorb H^+^ from water and act as the active sites for hydrogen evolution. An efficient electron transfer from the conduction band of ZnIn_2_S_4_ to NiS in which the hydrogen evolution occurs is crucial for the enhanced hydrogen evolution over the NiS/ZnIn_2_S_4_ nanocomposites since controlled experiments performed over a mixture of NiS and ZnIn_2_S_4_ shows a much lower photocatalytic activity under similar condition (Figure 5d). Therefore, the formation of a good junction between ZnIn_2_S_4_ and NiS via the two step hydrothermal processes is important for achieving the highly efficient NiS/ZnIn_2_S_4_ nanocomposites with enhanced photocatalytic hydrogen evolution activity.

## Conclusion

In summary, NiS/ZnIn_2_S_4_ nanocomposites were facilely synthesized via a two-step hydrothermal method. The as-prepared NiS/ZnIn_2_S_4_ nanocomposites showed highly enhanced photocatalytic performance for hydrogen evolution under visible light irradiation. The highest photocatalytic hydrogen evolution rate (104.7 μmol/h), which is even higher than that over Pt/ZnIn_2_S_4_ nanocomposite (77.8 μmol/h), was observed over an optimum NiS loading amount of 0.5 wt %. This work demonstrates a high potential of developing the environmental friendly, cheap non-noble-metal co-catalyst for semiconductor-based photocatalytic hydrogen evolution.

## Experimental

**Preparations.** All the reagents are analytical grade and used without further purifications. Hexagonal ZnIn_2_S_4_ powder was synthesized according to our previously reported method [16]. In a typical synthesis of 0.5 wt % NiS/ZnIn_2_S_4_ photocatalyst, 0.2 g ZnIn_2_S_4_, 2.8 mg nickel acetate and 0.9 mg thioacetamide (TAA) were dispersed in 70 mL of de-ionized water by stirring and ultra-sonication for 2 h. The resultant solution was transferred to a 100 mL Teflon liner, sealed in the stainless steel autoclave and heated at 120 °C for 4 h. After the autoclave was cooled to room temperature, the product was collected and washed with de-ionized water several times before it was dried at 60 °C to obtain the final product. Samples with different amount of NiS (0.2, 0.25, 1.0, 2.0 wt %) were prepared by using different amounts of nickel acetate and TAA precursor during the hydrothermal treatment at 120 °C, while keeping other conditions the same.

0.5 wt % Pt/ZnIn_2_S_4_ was prepared by a photo-deposited method using H_2_PtCl_6_·6H_2_O as the starting material. Pure NiS was prepared by hydrothermal using nickel acetate and TAA as precursors at 120 °C for 4 h.

**Characterizations.** X-ray diffraction (XRD) patterns were collected on a Bruker D8 Advance X-ray diffractometer with Cu Kα radiation. The transmission electron microscopy (TEM) and high-resolution transmission electron microscopy (HRTEM) images were measured by a JEOL model JEM 2010 EX instrument at an accelerating voltage of 200 kV. The powder particles were supported on a carbon film coated on a 3 mm diameter fine-mesh copper grid. A suspension in ethanol was sonicated and a drop was dripped on the support film. X-ray photoelectron spectroscopy (XPS) measurements were performed on a PHI Quantum 2000 XPS system with a monochromatic Al Kα source and a charge neutralizer. All of the binding energies were referred to the C 1s peak at 284.8 eV of the surface adventitious carbon. UV–visible diffraction spectra (UV–vis DRS) of the powders were obtained for the dry pressed disk samples using a UV–visible spectrophotometer (Cary 500 Scan Spectrophotometers, Varian). BaSO_4_ was used as a reflectance standard.

**Photocatalytic hydrogen evolution.** Photocatalytic hydrogen evolution experiments were carried out in a closed gas circulation and evacuation system fitted with a top Pyrex window. 50 mg of photocatalyst was dispersed in 100 mL of aqueous solution containing 0.5 M Na_2_SO_3_ and 0.43 M Na_2_S as sacrificial reagents. The suspension was irradiated with a 300 W Xe lamp equipped with a 420 nm cutoff filter to provide the visible light irradiation. The temperature of the reactant solution was maintained at room temperature by a flow of cooling water during the photocatalytic reaction. The amount of hydrogen evolved was determined with an on-line gas chromatography equipped with a TCD detector.
